# Relations of COVID-19-Related Stressors and Social Support With Chinese College Students' Psychological Response During the COVID-19 Pandemic

**DOI:** 10.3389/fpsyt.2020.551315

**Published:** 2020-10-30

**Authors:** Xiaoshan Li, Hou Wu, Feifei Meng, Li Li, Yitong Wang, Mingjie Zhou

**Affiliations:** ^1^School of Psychology, Jiangxi Normal University, Nanchang, China; ^2^Nanchang Institute of Technology, Nanchang, China; ^3^CAS Key Laboratory of Mental Health, Institute of Psychology, Chinese Academy of Sciences, Beijing, China; ^4^Department of Psychology, University of Chinese Academy of Sciences, Beijing, China

**Keywords:** PTSD, depression, anxiety, social support, COVID-19-related stressors

## Abstract

This study examines the main and interactive relations of stressors and social support with Chinese college students' psychological symptoms (e.g., anxiety, depression) during the COVID-19 pandemic. All the constructs are assessed by self-report in an anonymous survey during the pandemic outbreak. The results show that the number of stressors has a positive relation with psychological symptoms, and social support has a negative relation with psychological symptoms. In addition, social support serves as a buffer against the negative impact of stressors. These findings hold implications for university counseling services during times of acute, large-scale stressors. Specifically, effective screening procedures should be developed to identify students who experience large number of stressors and provide suitable psychological intervention for them.

## Introduction

It is well-established that stressful events (e.g., earthquakes and fatal diseases) have a significant impact on individual physical and mental health (1). However, the impact of large-scale stressors (e.g., infectious disease) on psychological adjustment in the general population is understudied, and we know little about how to improve psychological adjustment in the general Chinese population during the COVID-19 pandemic outbreak. The present study aims to examine the main and interactive relations of stressors and social support to individual psychological responses during the COVID-19 pandemic outbreak and holds the promise of information for counselors to prevent the negative psychological effects of the pandemic on the general college student population.

### Relation of Stress to Psychological Response

Lazarus and Folkman (2) define stress as “a particular relationship between the person and the environment that is appraised by the person as taxing or exceeding his or her resources and endangering his or her well-being” (p.19). To maintain biological homoeostasis during environmental or physiological challenges, our physiological coping mechanisms involving the hypothalamic-pituitary-adrenal axis, the autonomic nervous system, and the cardiovascular, metabolic, and immune systems protect the body from internal or external stress (3). A mild, brief, and controllable state of challenged homeostasis could actually be perceived as pleasant or exciting and could be a positive stimulus for emotional and intellectual growth and development (4). However, if the normal stress response occurs frequently, it is not self-limited, and if the individual does not adapt to a repeated stressor of the same type, adverse metabolic consequences occur. A large body of research literature has shown links between chronic and acute stress and short- or long-term psychological and physical disorders, such as anxiety, depression, and PTSD (5–7). Specifically, people who experience a large number of stressors related to infectious diseases tend to show more psychological symptoms (e.g., PTSD, depression) than those who experience fewer stressors (6, 8). The COVID-19-related stressors are those stressful events related with COVID-19 disease that might endanger their well-being, such as an important person being infected with COVID-19 or having to cancel a vacation due to the pandemic. Facing the COVID-19 pandemic, an individual might experience different COVID-19-related stressors for many other reasons. Thus, the first hypothesis is proposed as follows:

Hypothesis 1: Individuals who experience more COVID-19-related stressors might have more psychological symptoms than those who experience less.

### Relation of Social Support to Psychological Adjustment

The conservation theory of resources posits that both resource loss and resource gain are key predictors of psychological response, such as depression (9). Infectious diseases, such as with the COVID-19 pandemic, are often large scale and beyond individual control, and they lead to major resource loss. Social support is often regarded as an important compensation mechanism in buffering individual psychological responses when facing challenging environments (10). On the one hand, social support could be beneficial to individual mental health by providing the needed material and mental resources for dealing with life challenges. On the other hand, social support could improve individual psychological adjustment by enhancing the individual sense of control in dealing with stressful events. A vast body of research has demonstrated that the adequacy of social support is negatively related to the severity of psychological symptoms, such as depression (5, 11–13), and is positively related with well-being (14). A meta-analysis shows that social support is the strongest predictor of PTSD severity (15).

Social support is also regarded as a moderator in the relation between stressors and psychological outcomes (10). The moderating role of social support can be explained in the following ways. First, previous work suggests that perceived social support fosters feelings of belonging and security and a sense of control over the environment, which may enhance self-esteem, thus reducing the negative effects of stress on psychological adjustment (16–18). Second, research shows that social support could decrease the likelihood of maladaptive inferences about actual stressful life events by forming new schemas or revised schemas, which may attenuate the negative psychological outcomes of stressful events (19–21). Many studies show that people who perceive adequate social support find negative events to have fewer adverse consequences (e.g., anxiety, depression) than those who perceive little or no support (22–24). Thus, social support might moderate the stress-psychological symptom relation during the COVID-19 pandemic. The second hypothesis is proposed as follows:

Hypothesis 2: Social support (a) is a protective factor in predicting individual psychological symptoms and (b) could be regarded as a buffer in the relation between COVID-19-related stressors and psychological symptoms.

### The COVID-19 Outbreak as a Unique Context for Studying Stressors, Social Support, and Psychological Symptoms

As an unusual and contagious pneumonia, the COVID-19 pandemic is caused by a novel and highly transmissible coronavirus. It caused international concern for its speed of transmission and varying degrees of illness and is designated by WHO as a public health emergency of international concern (25). As of March 15, 2020, a total of 150,000 laboratory-confirmed cases and 5,720 deaths have been documented in 141 countries or areas (26), and mainland China is one of the most highly infected areas. Under this circumstance, the Chinese government officially stepped up pandemic prevention and control with stricter measures to prevent the virus from spreading (25). These measures include tracing contacts, strengthening traffic control in each city, forbidding mass gatherings, closing nonessential public places, deferring the opening time of schools, and replacing in-person teaching with online teaching. Despite the benefits, these strict measures may also create heavy psychological and emotional burdens on the general population. For example, an individual may be quarantined because of a suspected or confirmed infection or an individual might cancel a vacation trip because of the COVID-19 pandemic. In particular, the student population (e.g., college students) became susceptible to the pandemic due to its large population density and their immaturity. Research shows that individuals across samples (from the general public to healthcare workers) report significant psychological responses during the outbreak (27–29). Psychological responses include perceiving COVID-19-related fear, threat, and worry and experiencing symptoms of psychological disorder. Moreover, these studies report significant individual differences in psychological response to COVID-19-related stressors and in susceptibility to mental health problems during the pandemic. However, these studies mainly focus on general local residents who live in infected areas and front-line health care workers rather than the general population. Although these studies investigate the impact of COVID-19 disease on individual psychological responses (e.g., perceived stress to COVID-19 disease) and psychological disorders (e.g., depression) during pandemic outbreaks, the relationship between COVID-19-related stressors and psychological adjustment during pandemics and their underlying mechanisms have not been thoroughly investigated. Therefore, studying the impact of the COVID-19 pandemic holds the promise of information for counselors to prevent the negative psychological effects of pandemics on the general college student population.

## Method

### Participants

Data were collected between March 2 and March 15, 2020, using an anonymous, self-reported questionnaire. A total of 450 college students with different majors who participated in the curriculum of “Happy psychology” and “Mental health and education for primary and secondary school students” were invited to take part in our survey. The research material was sent to each student by email. The students were asked to return the completed survey as well as signed informed consent by the deadline. This study was conducted under the approval of the Moral and Ethics Committee of the School of Psychology, Jiangxi Normal University (Nanchang, China). Questionnaires with more than 15% of the items unanswered were excluded from the late analysis. A total of 431 survey responses were used in the following analysis. Of the respondents, 267 (61.9%) are female, and participant ages range from 18 to 22 (*M* = 19.1, SD = 0.92). Regarding their grade, 140 (32.5%) are freshmen; 197 (45.7%) sophomore; 54 (12.5%) junior, and 40 (9.3%) senior students. There are 33 (7.7%) students who lived in Hubei Province (the hardest hit areas) during the pandemic outbreak.

### Measurement

#### COVID-19-Related Stress

According to the stress definition of Lazarus and Folkman (2), stressors are events that might endanger one's well-being. Main et al. (6) found that the stressors related to infectious disease could be grouped into six categories during the SARS epidemic, including self-, family-, friend-, acquaintance-, information-, and other infectious disease–related events. Main's view on stressor categorization of infectious disease is supported in our interview survey during the COVID-19 pandemic. Therefore, we developed a checklist measure to assess participants' experience of COVID-19-related stressful events (stressors) based on Main's SARS-related stressor scale. These events are grouped into six categories: (a) self-related events (three items, e.g., “You have experience contacting someone with a confirmed COVID-19 case”), (b) family-related events (three items, e.g., “A member of your family is suspected of having COVID-19”), (c) friend-related events (three items, e.g., “A close friend of yours is diagnosed with COVID-19 and receives treatment”), (d) acquaintance-related events (three items, e.g., “Someone you know [not including your family or a close friend] has COVID-19-like symptoms [fever, coughing]”), (e) information-related events (two items, e.g., “You heard others talking about the severity and contagiousness of COVID-19”), and (f) other COVID-19-related events (two items, e.g., “You had to cancel your vacation because of the COVID-19 pandemic”). Participants indicated whether each event occurred since the COVID-19 pandemic outbreak. The total number of events endorsed across all categories was computed with a high score indicating that students experience more COVID-19-related stressors.

#### Anxiety

The Chinese version of the GAD-7 is a one-dimensional, self-reported scale that is used to assess the symptoms of anxiety in adults (30). The participants were asked to rate perceived symptom burden during the past 2 weeks between 0 (not at all) and 3 (nearly every day), resulting in a total score ranging from 0 to 21. Higher scores indicate that the students might have higher symptoms of anxiety during the pandemic. The Chinese version of the GAD-7 demonstrates good psychometric properties (31) with a Cronbach's α of 0.86 in the present study.

#### Depression

The PHQ-9 is widely used to measure depression severity in adults with one item for each of the nine depressive symptoms (32). The participants were asked to rate perceived symptom burden during the past 2 weeks between 0 (not at all) and 3 (nearly every day), resulting in a total score ranging from 0 to 27. Higher scores indicate that the students might have higher symptoms of depression during the pandemic. The PHQ-9 is translated into various languages (including Chinese) and yields robust reliability in adults (including Chinese samples) (31, 32). The Cronbach's α of the PHQ-9 is .85 in the present study.

#### PTSD

The Impact of Events Scale – Revised [IES-R; (33)] is used to measure PTSD symptoms. The IES-R consists of 22 items: eight for re-experiencing/intrusion symptoms, eight for avoidance symptoms, and six for hyper-arousal symptoms. Each item is rated on a 5-point Likert-type scale between 0 (not at all) and 4 (extremely), reflecting the extent to which the particular symptom is a problem for the respondent during the past 1 week. The IES-R is demonstrated to be a valid tool in diagnosing PTSD in a Chinese sample (33) with Cronbach's α of the three dimensions of the IES-R ranging from 0.85 to 0.91 in the present study.

#### Perceived Social Support

The perceived social support scale (34) is used to measure the individual's degree of satisfaction of the support received from family, close friends, and other-related persons (such as teachers and common students but excluding close friends and family members). The scale includes three items, and the participants were asked to rate each item scored on a four-point Likert scale ranging from “very satisfied” to “very dissatisfied.” Higher scores indicate greater satisfaction with the support from family members and others. The Cronbach's α of the perceived social support scale is 0.85 in the present study.

#### Control Variables

Previous research shows that sex, family residence (or the degree of exposure to disease), and age (or grade) are important influencing factors in predicting individual psychological adjustment during infectious disease (6, 29). Therefore, the variables of sex, grade, and family residence (Hubei vs. non-Hubei) are used as control variables in the present study.

### Data Analysis

Considering that the indicators of psychological symptoms might be correlated with each other, it is appropriate to test the relations between those outcome variables and their predictors simultaneously in one model (35). The multivariate general linear model is used to investigate the effect of predictors (e.g., COVID-19-related stressors, social support) on the following psychological symptoms (e.g., anxiety, depression, avoidance, intrusion, and hyper-arousal) while simultaneously controlling for the effect of sex, grade, and family residence. Before running the model, the parametric assumptions on data (linearity, multicollinearity, multivariate normality, and homogeneity of variances) and the potential presence of multivariate outliers were assessed by inspecting diagnostic plots and performing ad hoc statistical tests, such as the Shapiro–Wilk test for multivariate normality, the Box's *M* test for homogeneity of covariances, and the Mahalanobis distance test for multivariate outlier detection. With the exception of linearity and collinearity (pair-wise correlations among dependent variables ranged from 0.32 to 0.83, all *p* < 0.01), the results of which were acceptable, all other assumptions were more or less violated. However, the multivariate general linear analysis is robust enough to such violation when the sample size is large (36). After deleting the 22 multivariate outliers that were identified by the Mahalanobis distance method, all skewness values became low and acceptable (between 0 and 1). A separation score was finally calculated for each dependent variable (e.g., anxiety) as the ratio of its between-group variance and its within-group variance. The higher the score, the greater the separation between groups that a variable gives. Data exploration and statistical analyses were performed with SPSS 16.0.

## Result

The means, standard deviations, and zero-order correlation for the full sample are presented in Table 1. The results show that the number of COVID-19-related stressors is positively related to psychological symptoms (e.g., anxiety, depression, intrusion, avoidance, and hyper-arousal) (all *P* < 0.01), and social support is negatively related to the psychological symptoms above (all *P* < 0.01).

**Table 1:** Zero-order correlation of study variables for the full sample (*N* = 431).

**Variable**	***M ± SD***	**1**	**2**	**3**	**4**	**5**	**6**	**7**
1 Anxiety	3.85 ± 3.62	1						
2 Depression	5.73 ± 4.36	0.51^**^	1					
3 Avoidance	6.61 ± 5.77	0.48^**^	0.32^**^	1				
4 Intrusion	6.21 ± 5.12	0.60^**^	0.40^**^	0.75^**^	1			
5 Hyper-arousal	3.72 ± 3.84	0.65^**^	0.45^**^	0.73^**^	0.83^**^	1		
6 Social support	4.98 ± 1.26	−0.32^**^	−0.48^**^	−0.17^**^	−0.18^**^	−0.23^**^	1	
7 Stressors	2.63 ± 1.21	0.14^**^	0.19^**^	0.16^**^	0.18^**^	0.17^**^	0.05	0.1

*Stressors, COVID-19-related stressors*.

** *p < 0.01 (two-tailed test)*.

### One-way Multivariate Analysis

The effect of predictors and demographic variables on psychological symptoms was tested one by one. A multivariate general linear model was used for factors (e.g., sex, grade, family residence) and continuous variables (e.g., COVID-19-related stressors and social support scores). All indicators of psychological symptoms (including anxiety, depression, avoidance, intrusion, and hyper-arousal) were regarded as outcome variables and were entered into the multivariate general linear model simultaneously. Results show that all demographic variables and predictors are significantly related with psychological symptoms (all *P* < 0.05). Furthermore, the examination of η^2^ (eta squared) reveals small to medium effect sizes (from 0.03 to 0.11) with COVID-19-related stressors (η^2^ = 0.06) and social support (η^2^ = 0.11) showing a one-way moderate effect according to Cohen's criteria (37). In contrast analysis, significant linear (all *P* < 0.001), and quadratic (all *P* < 0.05) trends across the variables of COVID-19-related stressors and social support were found. Specifically, students who perceived high levels of COVID-19-related stressors (or social support) reported more (or less) psychological symptoms than did peers who perceived less, and hypothesis one is supported. The significant linear trends across the demographic variables (all *P* < 0.001) are also found in contrast analysis. That is, female students report more psychological symptoms than male students. Junior or senior students report more psychological symptoms than freshmen or sophomores. Students whose family residence is in Hubei province report more psychological symptoms than peers whose family residence is not in Hubei Province.

### Multiple Multivariate Analysis

To test the independent impact of COVID-19-related stressors and social support on psychological symptoms, all independent variables (COVID-19-related stressors, social support, sex, grade, and family residence) were entered into a multivariate general linear model together. As presented in Table 2, all variables are significantly associated with psychological symptoms (all *P* ≤ 0.05) and η^2^*s* were not much dissimilar from one-way multivariate analysis results. The effect size of COVID-19-related stressors and social support became a little bit smaller. Although the quadratic trend is not statistically significant, the linear trend continues to be statistical significant (for COVID-19-related stressor: Wilk's λ = 0.87, *F*_(25, 1, 491)_ = 2.54, *P* < 0.001, η^2^ = 0.05; social support: Wilk's λ = 0.76, *F*_(85, 1, 943)_ = 2.84, *P* < 0.001, η^2^ = 0.1).

**Table 2:** Multivariate general linear results with effect sizes (*N* = 409).

	**Wilks**	***F***	**Hypothesis df**	**Error df**	***P***	**η^**2**^**
COVID-19-related stressors	0.92	3.59	10	782	<0.001	0.05
Social support	0.84	7.74	10	782	<0.001	0.08
Sex	0.97	2.72	5	391	<0.05	0.03
Grade	0.96	3.24	5	391	<0.01	0.04
Family residence	0.98	2.23	5	391	<0.05	0.03

*Multivariate general linear was used to investigate our hypotheses, in which the indicators of anxiety, depression, avoidance, intrusion, and hyper-arousal are outcomes variables; both COVID-19-related stressors and social support are predictors; and sex, grade, and family residence are covariates*.

**Figure 1:**
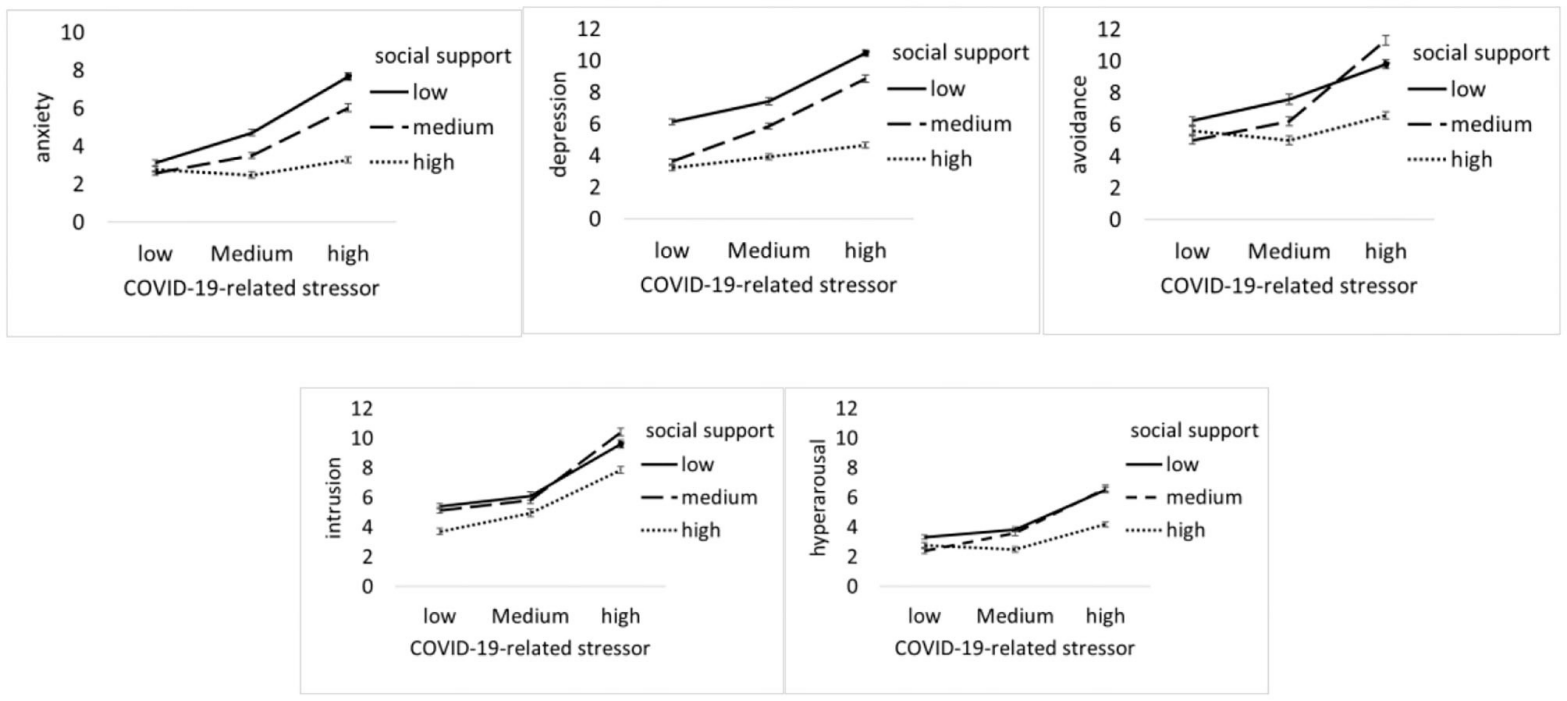
COVID-19-related stressor by social support. Unadjusted psychological symptoms (including anxiety, depression, avoidance, intrusion, and hyper-arousal) data are plotted; means and standards errors are displayed.

**Figure 2:**
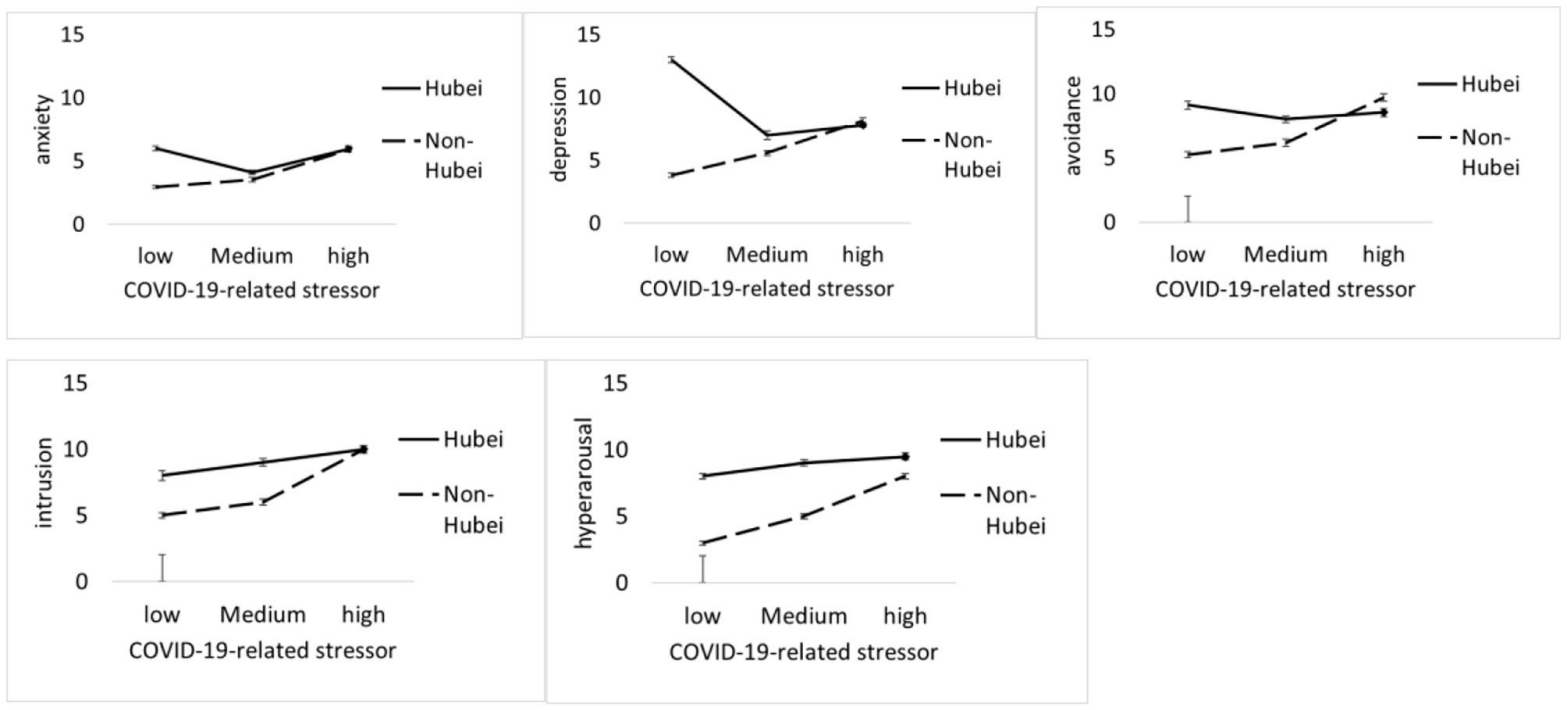
COVID-19-related stressor by family residence. Unadjusted psychological symptoms (including anxiety, depression, avoidance, intrusion, and hyper-arousal) data are plotted; means and standards errors are displayed.

In order to examine the interaction effect, a new multivariate general linear model was built, including COVID-19-related stressors, social support, sex, grade, and family residence as factors. In the new model, both COVID-19-related stressors and social support are classified as three groups: low (the scores below−1 SD from the mean), medium (the scores between −1 SD from the mean and +1 SD from the mean), and high (the scores above +1 SD from the mean). Two two-way interaction effects (e.g., family residence × COVID-19-related stress and social support × COVID-19-related stress) are significant. Hypothesis 2 is supported. Specifically, for social support, with the COVID-19-related stressors increased, the psychological symptoms (e.g., anxiety, depression, and avoidance) of students increased sharply at the lower level of the social support group; in contrast, the psychological symptoms of students increased relatively slowly at the high level of social support group (see Figure 1). For family residence, with the COVID-19-related stressors increased, the psychological symptoms of students increased sharply at non-Hubei province; in contrast, the psychological symptoms of students increase relatively slowly at Hubei province (see Figure 2).

Finally, separation scores show that COVID-19-related stressors are mostly associated with the symptoms of depression scale, followed by intrusion and anxiety symptoms; social support is mostly related with the symptoms of depression scale, followed by anxiety symptoms (see Table 3). In other words, the significant trend that is found in the multivariate general linear model, i.e., the differences of psychological symptoms in different levels of COVID-19-related stressors (or social support) groups, is mainly due to the large between-group differences in depression and anxiety mean scores. In addition, similar results are found for demographic variables. That is, the demographic variable (e.g., sex, grade, family residence) differences on individual psychological symptoms are mainly due to the large between-group differences in depression and anxiety mean scores, too (See Table 3).

**Table 3a:** Separation values for each psychological symptom.

**Variables**	**COVID-19-related stressors**			**Social support**		
	**Within-group variance**	**Between-group variance**	**Separations^**a**^**	**Within-group variance**	**Between-group variance**	**Separations^**a**^**
Anxiety	11.2	97.38	8.69	11.2	141.45	12.63
Depression	14.5	147.38	10.17	14.5	521.47	35.96
Avoidance	30.83	190.23	6.17	30.8	140.51	4.56
Intrusion	23.79	215.88	9.07	23.8	106.98	4.5
Hyper-arousal	13.35	91.31	6.84	13.3	65.59	4.92

a *Calculated as the variable between-groups variance divided by its within-groups variance*.

**Table 3b:** 

**Variables**	**Sex**			**Grade**			**Family residence**		
	**Within-group variance**	**Between-group variance**	**Separations^**a**^**	**Within-group variance**	**Between-group variance**	**Separations^**a**^**	**Within-group variance**	**Between-group variance**	**Separations^**a**^**
Anxiety	11.2	83.34	7.4	11.2	69.41	6.16	11.2	79.51	7.09
Depression	14.5	139.18	9.51	14.5	55.19	3.81	14.5	60.04	4.28
Avoidance	30.83	14.99	0.48	30.83	112.49	3.61	30.83	85.99	2.79
Intrusion	23.79	58.76	2.49	23.79	91.92	3.86	23.79	49.79	2.09
Hyper-arousal	13.35	81.52	6.04	13.35	49.79	3.71	13.35	5.86	0.44

a *Calculated as the variable between-groups variance divided by its within-groups variance*.

## Discussion

As a global public emergency issue, the COVID-19 pandemic has caused great suffering for those people who live in infected areas. Although there has been some research focusing on the influence of the pandemic on the psychological adjustment of patients and front-line healthcare workers, we know little about the influence of the COVID-19 pandemic on general population (e.g., college students). Therefore, studying the relationship between COVID-19-related stressors and Chinese college students' psychological adjustment during pandemics and their underlying mechanism could enrich our understanding about the impact of the pandemic on the general population. Our findings also could hold the promise of information for counselors to prevent the negative psychological effects of pandemics on the general college student population.

### Main Effects of COVID-19-related Stressors and Social Support on Psychological Symptoms

Correlation analysis shows that the number of COVID-19-related stressors has a positive relation with psychological symptoms (including anxiety, depression, and PTSD symptoms), which is consistent with previous findings on individual adjustment during other infectious disease pandemics (e.g., SARS) in the general public sample (6). That is, during an acute, large-scale pandemic, such as the COVID-19 pandemic, even among individuals who are not directly contaminated with the disease, the psychological impact of the pandemic on the general population is significant. In addition, the negative relation found between social support and psychological symptoms is consistent with previous studies in which social support could be a protective predictor in stressful events, such as SARS, swine flu, or Middle East respiratory syndrome (MERS) (7, 38, 39).

### Interactions Between Stress and Social Support or Family Residence in Predicting Symptoms

COVID-19-related stressors and social support interact with one another in predicting anxiety, depression, and the avoidance of PTSD. That is, the negative effect of COVID-19-related stressors on individual psychological symptoms is larger at a low level of social support than those at a high level. The symptoms of depression, anxiety, and avoidance could explain most of the interaction effect. This suggests that social support serves as a buffer against the impact of COVID-19-related stressors on psychological symptoms during the COVID-19 pandemic. For PTSD, comparing with the subscales of intrusion and hyper-arousal, the symptom of avoidance is more likely explained by the interaction of COVID-19-related stressors and social support. A possible reason might be that social support could provide enough resources in dealing with stressors and decrease the usage of avoidance coping strategies during acute, uncontrollable circumstances. However, the symptoms of intrusion and hyper-arousal might be common psychological responses related to biological mechanisms during the pandemic, which could be less affected by environmental factors (e.g., social support). For family residence, comparing with the students whose family residences are in Hubei province (the most affected area), the psychological symptoms of those students whose family residences are not in Hubei province are more likely affected by COVID-19-related stressors. This could be explained by the “psychological typhoon eye” effect, in which the impact of stress on psychological symptoms could be reduced because of the low level of posttraumatic event concern in extremely devastated areas (40).

### Sex and Demographic Differences

Consistent with previous research (38), women tend to report higher psychological symptoms (e.g., anxiety, depression, and hyper-arousal symptoms) than men during a large-scale and uncontrollable pandemic. The possible reason might be that women tend to be more sensitive to external threat due to their biological factors (41, 42). For grade, students in a higher grade (e.g., junior or senior) reported increased symptoms of anxiety, depression, and PTSD than those students with lower grade (e.g., freshmen or sophomores). A possible reason might be that, compared with freshmen and sophomores, senior or junior students may experience more stress from graduation and looking for a job. With regard to other demographic variables, students whose family residence was in Hubei Province (the most infected area) reported higher anxiety and depression symptoms than did peers whose family residence was not in Hubei Province. The possible reason might be that, in an infected area, such as Hubei Province, students themselves and their family members or friends experience a greater threat from COVID-19. Therefore, in such cultures emphasizing family and relationships, students from the most infected areas tend to experience more psychological symptoms during a pandemic.

### Implications of the Study for University Counseling Services

As one of the few studies on the relations of the stressors and social support with psychological symptoms among college students during the COVID-19 pandemic, this study has important implications for university counseling services during acute, large-scale stressors, such as an infectious disease outbreak or natural disaster. First, given the study findings, even students who are not directly affected by COVID-19 report significant numbers of COVID-19-related stressors and psychological symptoms during the pandemic. It is crucial that university campuses develop and implement effective screening procedures to closely monitor students' exposure to stressors and mental health adjustment. Second, the present study shows that social support is negatively related to anxiety, depression, and PTSD symptoms and served as a buffer against the negative effect of COVID-19-related stressors. In Asian culture, which is concentrated on family or kinship support (43), social support is a crucial resource for college students to deal with stressors during pandemic outbreaks. Third, the present study suggests that female students, students at higher grade levels, and those students whose family residences are located in pandemic-affected areas show vulnerability in the face of an infectious disease and experience more psychological symptoms. These students should receive more attention from university counseling services during pandemic outbreaks. In addition, the checklist measure for COVID-19-related stressful events developed in the present study can be modified to monitor students' exposure to disaster-related stressors, and students who are exposed to a large number of stressors should be identified to receive some preventive services.

### Limitations

The study has several limitations. First, because all the constructs were assessed by self-report, the estimated relations among stressors, social support, and psychological symptoms might be biased by the reporter effect. Future research should consider a longitudinal design or use a multimethod to examine the role of stressors and social support in individuals' psychological symptoms during an acute, large-scale pandemic outbreak. Second, there has been some speculation that Asian cultures tend to emphasize family and relations more than Western cultures (43). It is possible that the interaction of these stressors and social support might not generalize to Western populations. Future research should test culture as a moderator of the relation between social support and adjustment using cross-cultural comparative samples (6). Third, although the sample used in the present study consists of students with different majors and is a representative sample of the college population to some degree, all of the participants were enrolled in two courses offered by the School of Psychology in one tertiary education institute, which might result in a bias in sampling. Future research could investigate the relations among the studied constructs with a large representative college student sample. In addition, research has shown that different types of stressors might have different degrees of influence on individual mental health (44). For example, some COVID-19-related stressors (e.g., “One of your family members has a confirmed COVID-19 case”) might have a larger impact on individual mental health than other stressors (e.g., “You have to cancel a vacation trip due to the pandemic”). The impact of the pandemic on psychological adjustment might be related to individual personality traits (e.g., coping) (20). For example, individuals who tend to use adaptive coping frequently might experience less psychological symptoms than those who use less adaptive coping during a pandemic outbreak. Therefore, future research could investigate the impact of stressors of different types or personality traits on individual psychological symptoms during pandemics.

## Data Availability Statement

The raw data supporting the conclusions of this article will be made available by the authors, without undue reservation.

## Ethics Statement

The studies involving human participants were reviewed and approved by the Moral and Ethics Committee of the School of Psychology, Jiangxi Normal Univerity (Nanchang, China). The patients/participants provided their written informed consent to participate in this study.

## Author Contributions

XL carried out the concepts, design, data acquisition, analysis, and manuscript editing. MZ carried out the concepts, design, and manuscript editing. HW carried out the design, data acquisition. FM carried out the design and data acquisition. YW carried out the data aquisition. LL carried out the data aquisition. All authors contributed to the article and approved the submitted version.

## Conflict of Interest

The authors declare that the research was conducted in the absence of any commercial or financial relationships that could be construed as a potential conflict of interest.
